# Endoscopic intermuscular dissection in the management of a rectal neuroendocrine tumor

**DOI:** 10.1055/a-2139-4310

**Published:** 2023-08-21

**Authors:** Suhuan Liao, Bo Li, Longbin Huang, Qiuping Qiu, Guang Yang, Jianzhen Ren, Silin Huang

**Affiliations:** Department of Gastroenterology, South China Hospital, Medical School, Shenzhen University, GuangDong, China

A 49-year-old man was admitted to our hospital for endoscopic resection of a subepithelial lesion located in the lower rectum ( Fig. 1 a ). The lesion was incidentally discovered during routine screening colonoscopy. Endoscopic ultrasound confirmed that the lesion originated from the submucosal layer ( Fig. 1 b ). Positron emission tomography with 2-deoxy-2-[fluorine-18]fluoro-D-glucose/computed tomography + speckle reduction imaging (18F-FDG PET/CT + SRI) suggested that the lesion was a neuroendocrine tumor (NET) with high expression of growth inhibitor receptors, without evidence of lymphatic or organ metastasis ( Fig. 2 ). The possibility of endoscopic resection was discussed with the patient, and subsequently, endoscopic intermuscular dissection (EID) was performed ( Video 1 ).

**Fig. 1:**
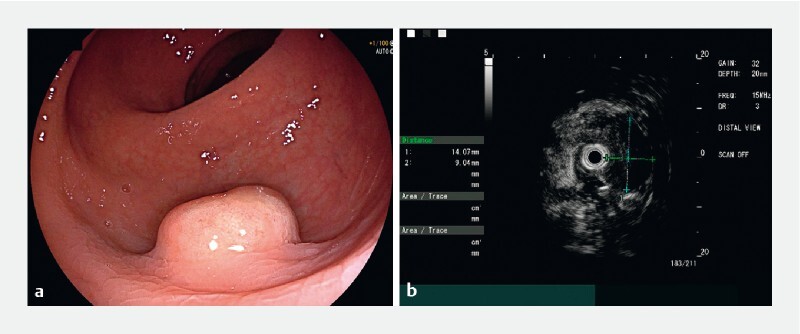
Colonoscopy and endoscopic ultrasound. **a** Colonoscopy revealed a subepithelial rectal lesion measuring approximately 14 mm in diameter. The lesion exhibited a yellowish appearance on the surface, with well-defined borders. **b** Endoscopic ultrasound showed a hypoechoic nodule originating from the submucosa, with clear border and uniform internal echogenicity, measuring approximately 14 × 6 mm in size.

**Fig. 2:**
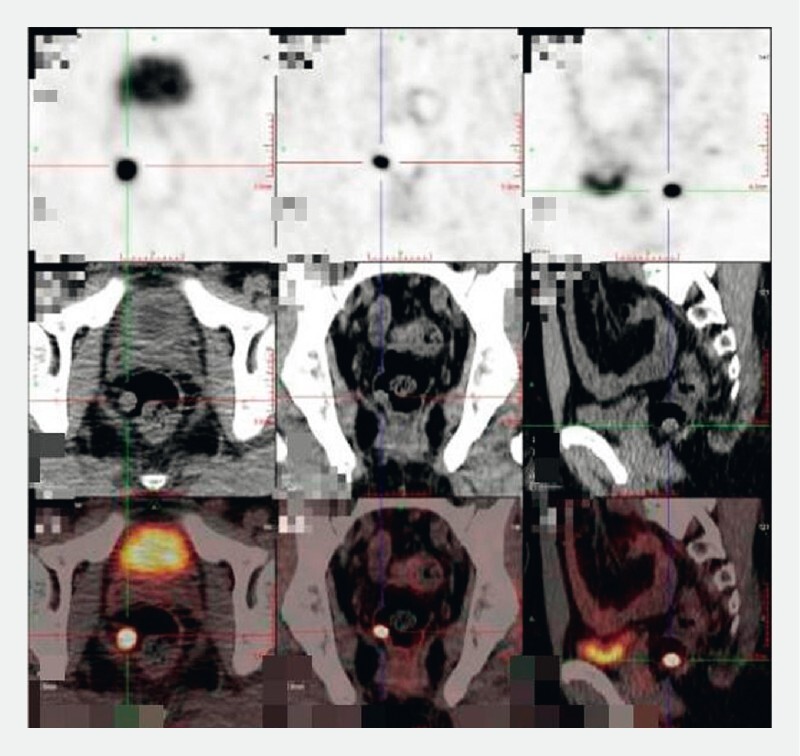
Positron emission tomography with 2-deoxy-2-[fluorine-18]fluoro-D-glucose/computed tomography + speckle reduction imaging (18F-FDG PET/CT + SRI) scan showed a soft tissue nodule in the lower rectum. The imaging revealed a significant increase in DOTATATE uptake and no increased FDG uptake, indicating a neuroendocrine tumor with high expression of growth inhibitor receptors.

**Video 1** Endoscopic intermuscular dissection in the management of a rectal neuroendocrine tumor.


First, the perimeter of the lesion was marked using soft tip coagulation, and submucosal injection was performed with a mixture of 0.9 % sodium chloride and indigo carmine ( Fig. 3 a ). Then, a circumferential incision was performed outside the markers, followed by submucosal dissection to expose the muscle layer ( Fig. 3 b ). An ST hood was attached to the tip of the endoscope, and the muscle fibers of the circular part of the muscle layer were cut off to gain access to the intermuscular space, exposing the longitudinal muscle layer ( Fig. 3 c ). Dissection was then continued in the intermuscular space until the tumor was resected ( Fig. 3 d ). After careful hemostasis, the defect was sutured with metal clips ( Fig. 3 e ). Finally, the specimen was stretched and immobilized for histopathologic evaluation ( Fig. 3 f ).

**Fig. 3:**
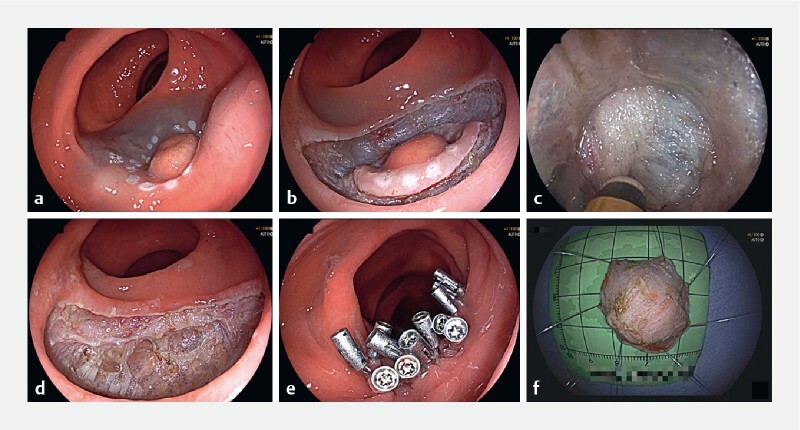
Endoscopic intermuscular dissection. **a** Lesion marking and submucosal injection. **b** Circumferential incision beyond the markers. **c** Intermuscular dissection: en bloc lesion removal and retrieval. **d** Postoperative defect. **e** Closure of the defect with metal clips. **f** Resected tumor.

There were no complications after the procedure, including perforation, bleeding, or fever. The patient was discharged after 72 hours and reported no discomfort during the follow-up period. Histopathological analysis ( Fig. 4 a–d ) revealed complete resection of a highly differentiated NET. The lesion was located within the circumferential muscle layer of the intestinal wall and showed local infiltration into the proximal longitudinal muscle layer. No tumor tissue was observed at the resection margins.

**Fig. 4:**
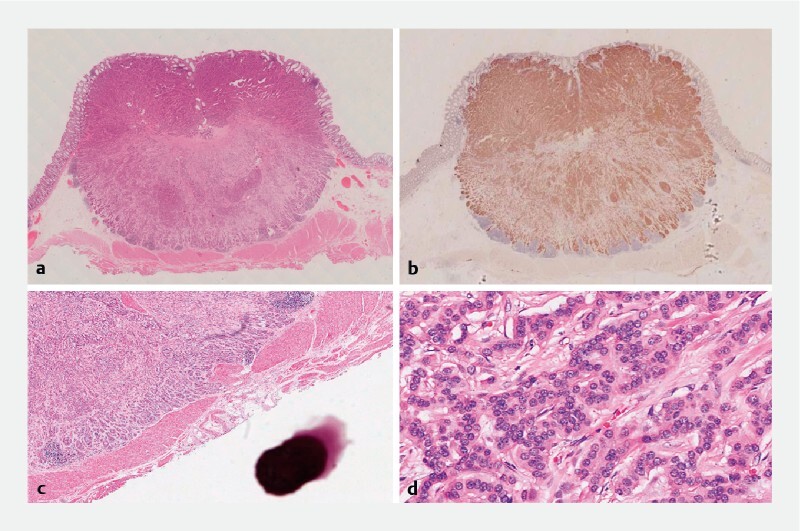
Histological findings. **a** Low-power field (hematoxylin and eosin [H&E]), showing a submucosal tumor extending into the submucosal layer. **b** Low-power field, positive staining observed with Syn stain. **c** Low-power field (H&E), illustrating tumor infiltration into the circular muscle layer, accompanied by resection of the circular muscle layer. **d** High-power field (H&E staining), demonstrating cells with round nuclei and pale cytoplasm arranged in a ribbon-like pattern.

In recent years, there has been increased detection of rectal NETs, which exhibit high heterogeneity and malignant potential. After detection, the recommended approach is generally endoscopic or surgical resection 1 . However, in some cases, positive vertical margins can still be observed following endoscopic mucosal resection or endoscopic submucosal dissection.

A study on rectal NETs < 16 mm without metastasis demonstrated that endoscopic submucosal dissection with myectomy yielded a higher rate of histological complete resection 2 . Additionally, reports have shown the feasibility and safety of EID for T1 rectal cancer 3 4 . Our case suggests that this technique can also be applied to rectal NETs with a larger diameter (> 10 mm) to ensure the negativity of the vertical margin.

Endoscopy_UCTN_Code_TTT_1AQ_2AD

## Notes

Endoscopy E-Videos

https://eref.thieme.de/e-videos
*E-Videos* is an open access online section of the journal *Endoscopy* , reporting on interesting cases and new techniques in gastroenterological endoscopy. All papers include a high-quality video and are published with a Creative Commons CC-BY license. Endoscopy E-Videos qualify for HINARI discounts and waivers and eligibility is automatically checked during the submission process. We grant 100% waivers to articles whose corresponding authors are based in Group A countries and 50% waivers to those who are based in Group B countries as classified by Research4Life (see: https://www.research4life.org/access/eligibility/ ). This section has its own submission website at https://mc.manuscriptcentral.com/e-videos
